# Quality of cancer treatment care before and after a palliative care pathway: bereaved relatives’ perspectives

**DOI:** 10.1136/spcare-2023-004495

**Published:** 2023-11-16

**Authors:** Annemieke van der Padt - Pruijsten, Maria BL Leys, Esther Oomen-de Hoop, Carin C D van der Rijt, Agnes van der Heide

**Affiliations:** 1 Internal Medicine, Maasstad Hospital, Rotterdam, The Netherlands; 2 Medical Oncology, Erasmus MC Cancer Institute, Rotterdam, The Netherlands; 3 Department of Public Health, Erasmus MC, University Medical Center Rotterdam, Rotterdam, The Netherlands

**Keywords:** cancer, end of life care, bereavement, advance care planning, hospital care

## Abstract

**Objective:**

Appropriate communication between healthcare providers and patients and their families is an essential part of good (palliative) care. We investigated whether implementation of a standardised palliative care pathway (PCP) facilitated communication, that is, aspects of shared decision-making (SDM), including advance care planning (ACP) conversations and satisfaction with care as experienced by bereaved relatives of patients with advanced cancer.

**Methods:**

We conducted a prospective preintervention and postintervention study in a hospital. Questionnaires were sent to relatives of patients who died between February 2014 and February 2015 (pre-PCP period) or between November 2015 and November 2016 (post-PCP period). Relatives’ perceptions on communication and satisfaction with care were assessed using parts of the Views of Informal Carers—Evaluation of Services and IN-PATSAT32 Questionnaires.

**Results:**

195 (46%) and 180 (42%) bereaved relatives completed the questionnaire in the pre-PCP and post-PCP period, respectively. The majority of all patients in both the pre-PCP period and the post-PCP period had been told they had an incurable illness (92% and 89%, respectively, p=0.544), mostly in the presence of a relative (88% and 85%, respectively, p=0.865) and had discussed their preferences for end-of-life (EOL) treatment (82% and 76%, respectively, p=0.426). Bereaved relatives were reasonably satisfied with the received hospital care in both groups.

**Conclusions:**

We found no overall effect of the PCP on the communication process and satisfaction with EOL care of bereaved relatives. Before the use of the PCP bereaved relatives already reported favourably about the EOL care provided.

**WHAT IS ALREADY KNOWN ON THIS TOPIC**

- Early integration of palliative and oncology care is important in order to comply with patients’ preferences for medical treatment and care.
- Shared decision-making (SDM) is one of the key elements of patient-centred palliative care and requires discussion of medical information and patients’ values and preferences.
- (Bereaved) relatives’ perspectives can inform research on quality of care.

**WHAT THIS STUDY ADDS**

- Use of a standardised palliative care pathway may be beneficial for the quality of hospital care for patients with advanced incurable cancer, but does not necessarily affect relatives’ satisfaction with care.
- Relatives are aware of challenges with the exchange of information.
- Many bereaved relatives appreciate an aftercare discussion.

**HOW THIS STUDY MIGHT AFFECT RESEARCH, PRACTICE OR POLICY**

- Improvement of information exchange between healthcare professionals is needed, since bereaved relatives were least satisfied with this aspect of care.
- To optimise the quality and consistency of bereavement care, hospitals should routinely offer aftercare discussions to bereaved relatives, especially if patients die in the hospital.

## Introduction

 For patients with advanced, incurable cancer early integration of palliative and oncology care is important in order to be timely able to comply with their preferences for medical treatment and care.^1 2^ Shared decision-making (SDM) is one of the key elements of patient-centred palliative care.^2^,^5^ In SDM, patients with advanced, incurable, cancer may weight the possible benefits of anticancer treatment and potential prolongation of life versus the risk of complications with substantial deterioration of quality of life. Moreover, SDM includes advance care planning (ACP), that is, discussion of preferences for future treatment and care.^6^ Making decisions about appropriate treatment requires discussion of medical information (eg, diagnosis, prognosis, treatment options) and patients’ values and preferences.^7^ Facilitators for SDM are, among others, a positive patient–physician interaction to ensure that patients trust their physicians and feel free to express their preferences, and involvement of family members and/or friends.^2 5^ Furthermore, information exchange between healthcare professionals, including information about ACP conversations, is important for patients' satisfaction and continuity of care.^8^

To support healthcare professionals who are not specialised in palliative care in integrating palliative care in oncology care, we developed a standardised digital palliative care pathway (PCP). This structured electronic medical checklist aims to support healthcare professionals in exploring patients’ values, needs and preferences, discussing possible interventions and coordination of (future) care, and documenting these discussions and decisions. The PCP includes guidance on identifying patients who might benefit from palliative care, by using the surprise question (‘Would you be surprised if this patient died within the next 12 months?’). After opening the PCP, various prompts guide the physician in exploring patients’ needs in all palliative care dimensions: physical, psychosocial and/or of spiritual nature. Furthermore, the PCP facilitates involvement of family and relatives and coordination of care. This coordination of care is facilitated by suggesting communication with the patient’s general practitioner and involvement of a palliative care team, pain team, social worker, psychologist and/or spiritual counsellor (online supplemental file 1).^9^ Using this PCP resulted in fewer medical interventions (including anticancer treatments), possibly indicating increased awareness among physicians of patients' impending death.^10^

The effect of complex interventions such as early integration of palliative care in oncology care is mainly studied by assessing the use of medical care at the end of life (EOL; eg, emergency room visits, used chemotherapy) or patients’ quality of life.^11^,^13^ Patients’ and (bereaved) relatives’ perspectives, and their satisfaction about care are also important outcome measures in research on quality of care.^14^,^20^ However, whether early integration of palliative care in oncology care affects the quality of palliative and EOL care has barely been studied.^21 22^ We investigated whether implementation of the PCP facilitated communication, that is, SDM, including ACP conversation, and satisfaction with care at EOL, as experienced by bereaved relatives.

## Methods

### Study design and population

This preintervention and postintervention study was part of a project investigating the effects of implementing a standardised PCP for patients with advanced cancer in a large teaching hospital in The Netherlands. Data were collected concerning adult patients who had been treated at the inpatient and/or outpatient clinic of the Departments of Oncology/Haematology and Lung Diseases and who died between February 2014 and February 2015 (pre-PCP period) or between November 2015 and November 2016 (post-PCP period). Details of this study have been reported elsewhere.^9 10^

Four weeks after a patient’s death, a letter was sent to the home address of the patient with our condolences and an advance notice about a survey studying the quality of and satisfaction with care at EOL as perceived by bereaved relatives. The questionnaire with further information about the survey was sent to the bereaved relatives 10–12 weeks after the patient’s death. Relatives who did not wish to participate were asked to voluntarily disclose their reason for non-participation on the front page of the questionnaire and return that page.

### Measurements

A questionnaire comprising 73 items was developed (online supplemental file 2). Sociodemographic characteristics of the patients and their relatives included gender, relationship, religion and level of education. The quality of communication between patients, their relatives and healthcare professionals in the last 3 months of life was measured using relevant items of the questionnaire developed by Witkamp *et al* and from the *Views of Informal Carers—Evaluation of Services (VOICES*) Questionnaire.^19 20^ Questions from the VOICES Questionnaire were translated into Dutch and back into English according to the *European Organisation for Research and Treatment of Cancer* (EORTC) guidelines for translating questionnaires.^23^ The questionnaire was pilot-tested among a mixed group of 13 persons (age between 31 and 66 years; educational level from low to high International Standard Classification of Education (ISCED); both healthcare professionals and lay people). They understood the questions and experienced no difficulties in answering them.

The *EORTC IN-PATSAT32* Questionnaire was used to measure relatives’ satisfaction with hospital care.^14^ This questionnaire includes 11 multi-item and 3 single-item scales (32 items in total) on the quality of care provided by hospital doctors and nurses, as well as other aspects of the quality of hospital care. Answers are given on a five-point Likert scale (poor/fair/good/very good/excellent) and scores are standardised through linear transformation to a 0–100 scale. A higher score implies that relatives were more satisfied with care.

The last question in the questionnaire was an open text box where participants could add comments or ask for support if necessary.

### Statistical analyses

Participants in this study were relatives of patients who were included in the study in either the pre-PCP or the post-PCP period; in the post-PCP period patients and their relatives were included irrespective of whether the PCP had been used (ie, the intention-to-treat principle was applied). The statistical significance of differences in patients’ and relatives’ characteristics and outcome measures between the pre-PCP and post-PCP period was tested using Mann-Whitney U tests for continuous variables, χ^2^ or Fisher’s exact tests for categorical variables, and χ^2^ tests for trends for ordinal variables. A power analysis was not performed, since the study concerned a secondary analysis of data from a larger study. A per-protocol analysis was carried out where pre-PCP experiences of bereaved relatives were compared with the experiences of only those relatives of patients in the post-PCP period for whom the PCP had actually been used.

## Results

**Figure 1:**
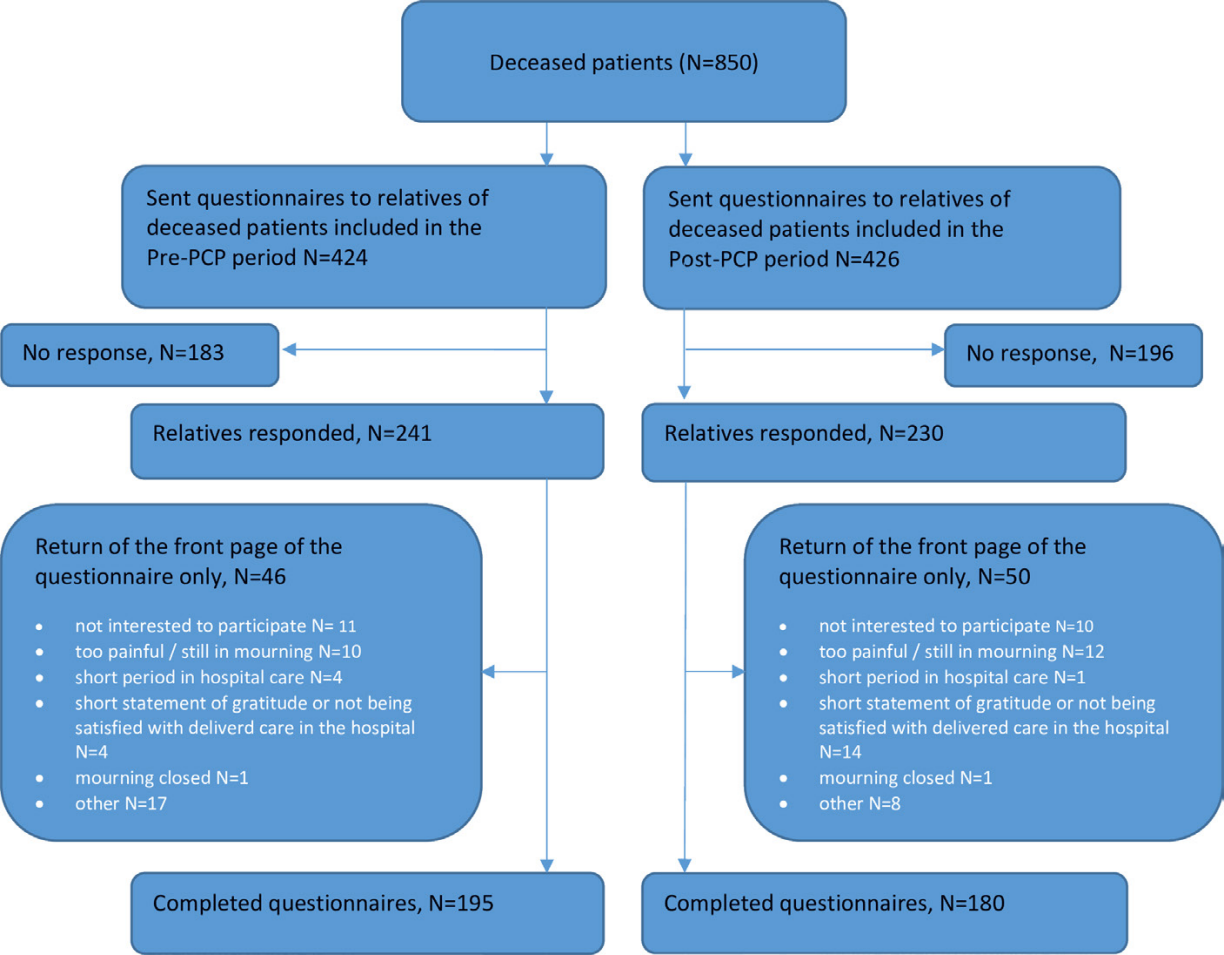
Flow chart of inclusion.

Questionnaires were sent to 424 relatives in the pre-PCP and 426 relatives in the post-PCP period. In the pre-PCP period, 241 (57%) relatives responded, of whom 46 (11%) filled out the front page only and 195 (46%) completed the questionnaire (figures 1). In the post-PCP period, 230 (54%) relatives responded, of whom 50 (12%) filled out the front page only and 180 (42%) completed the questionnaire. The most common reasons for not participating were not interested to participate; too painful/still in mourning; mourning closed; only a short period of in-hospital care. Some relatives wrote a short statement of gratitude or of not being satisfied with delivered care in the hospital on the front page of the questionnaire (figure 1). In 105 (58%) of the 180 post-PCP patients whose relatives had completed the questionnaire, the PCP had been used in the last phase of their life.

### Characteristics of patients and relatives

The mean age of the patients whose relatives completed the questionnaires was 71 years in the pre-PCP period and 73 years in the post-PCP period (p=0.042); somewhat more than half of all patients were male (58% and 59%, respectively). Gastrointestinal cancer was the most common primary cancer in both groups (34% and 33%, respectively). The majority of patients was married or living with a partner (76% and 77%, respectively) and had children (81% and 86%, respectively). Of all patients, one-third were religious, of whom a small part were Islamic (1% and 3%, respectively). Finally, most patients died outside the hospital and home was the most common place of death (45% and 42%, respectively). The relatives participating in the study had a mean age of 64 and 62 years, respectively, and were predominately the patient’s partner (70% and 59%, respectively) and in good health (62% and 58%, respectively) (table 1). No significant differences regarding the characteristics of relatives were found between the preintervention and postintervention periods. Similar results were found in the per-protocol analyses.

**Table 1:** Characteristics of the patients and their relatives in the pre-PCP period and the post-PCP period

	Pre-PCP(n=195)	Post-PCP(n=180)	P value
**Patients**			
Age at death (years) (mean SD)	71.0 (10.3)	72.9 (11.0)	0.042
Gender			
Male	113 (58)	106 (59)	0.854
Primary cancer			
Gastrointestinal	66 (34)	59 (33)	0.209
Lung	65 (32)	44 (23)	
Urogenital	24 (12)	28 (16)	
Haematological	22 (11)	31 (16)	
Breast	15 (7)	21 (11)	
Other	10 (5)	11 (6)	
Marital status			
Married/living with a partner	148 (76)	138 (77)	0.979
Widowed	31 (16)	28 (16)	
Other	15 (8)	13 (7)	
Children			
Yes	158 (81)	155 (86)	0.254
Living situation			
Alone	40 (21)	30 (17)	0.079
With partner	137 (70)	120 (67)	
Other	17 (9)	29 (16)	
Education^*^			
Low (ISCED level 1–2)	74 (38)	61 (34)	0.163
Intermediate (ISCED 3–4)	77 (39)	75 (42)	
High (ISCED 5–6)	32 (16)	36 (20)	
Other	7 (4)	1 (1)	
Religion			
Yes	71 (36)	71 (39)	0.681
Catholic/Protestant	58 (30)	59 (33)	
Islamic	1 (1)	3 (2)	
Other	11 (6)	9 (5)	
Place of death			
Patient’s own home	95 (49)	75 (42)	0.736
Relatives’ home	9 (5)	10 (6)	
Hospital	49 (25)	45 (25)	
Hospice	26 (13)	25 (14)	
Care home/nursing home	13 (7)	14 (8)	
Other	2 (1)	5 (3)	
**Relatives**			
Age (years) (mean SD)	63.6 (11.8)	62.2 (13.8)	0.502
Gender			
Female	119 (61)	106 (59)	0.629
Relation			
Partner/spouse of patient	137 (70)	106 (59)	0.096
Child (in law) of patient	40 (21)	56 (31)	
Other	17 (9)	16 (9)	
General health			
Very good	22 (11)	28 (17)	0.668
Good	123 (62)	105 (58)	
Average	35 (18)	29 (16)	
Good days/bad days	15 (8)	13 (7)	
Bad	2 (1)	4 (2)	

The number of missings varied between n=0–5 in the pre-PCP and n=0–7 in the post-PCP period.

* Education levels are categorised according to International Standard Classification of Education guidelines.

ISCEDInternational Standard Classification of EducationPCPpalliative care pathway

### Communication

No significant differences were found in relatives’ appreciation of communication characteristics between the pre-PCP and post-PCP period. According to relatives, most patients had been told they had an incurable illness in the presence of a relative (88% and 85%, in pre-PCP and post-PCP period, respectively) and most patients had discussed their preferences EOL treatment (82% and 76%, respectively). Relatives also reported that 14% of the patients in the pre-PCP period and 13% in the post-PCP period had needed more discussion about their preferences; 12% and 13% of the relatives, respectively, were not sure whether the patients had needed more discussion. For more than half of the patients in both groups the message of having an incurable disease had been discussed more than 3 months before death. Most relatives (79% and 79%, respectively) had been able to find out all they wanted to know about the illness of their loved ones, but for 25% and 29% of the relatives, respectively, more detailed information had been desirable. In both periods, most relatives had been involved with decisions about their loved one’s care and were satisfied with their involvement. Furthermore, the majority had been told their loved one was likely to die (86% and 82%, respectively), with two-thirds being satisfied with how this was told (68% and 71%, respectively) (table 2). In the per-protocol analysis, more relatives had been told their loved ones were likely to die in the post-PCP period compared with the pre-PCP period (86% and 90% respectively, p=0.042).

**Table 2:** Communication end-of-life

	Pre-PCP (n=195)	Post-PCP (n=180)	P value
	n (%)	n (%)	
Patient was told he/she had an incurable illness*	0.544
Yes, by a physician in the hospital (medical specialist/ward physician)	173 (92)	154 (89)	
Yes, by a family doctor or physician in a nursing home	6 (3)	6 (3)	
No	9 (5)	11 (6)	
Relative was present at the time of this message/discussion	171 (88)	153 (85)	0.865
How long before death the patient was told of his/her incurable illness	0.481
More than 12 months before death	53 (27)	55 (31)	
3–12 months before death	54 (28)	46 (26)	
1 week–3 months before death	65 (33)	57 (32)	
Less than a week before death	13 (7)	6 (3)	
Patient had discussed preference for EOL medical treatment with†:	160 (82)	137 (76)	0.426
Partner	112 (57)	90 (50)	0.517
Children and/or other family members and/or friends	97 (50)	104 (58)	
Medical specialist/family doctor/physician in a nursing home/nurse	130 (67)	113 (63)	
Patient had needed more discussion regarding his/her preferences for EOL medical treatment		0.866
No	136 (70)	122 (68)	
Yes	27 (14)	23 (13)	
Don’t know	23 (12)	24 (13)	
Relative had been able to find out all he/she wanted to know about his/her loved one’s illness and how it would probably affect him/her during the illness	154 (79)	143 (79)	0.641
Relative would have liked to receive more detailed information	48 (25)	52 (29)	0.220
Relative’s involvement with decisions about his/her loved ones’s care	0.186
Very involved	157 (81)	150 (83)	
Fairly involved	29 (15)	19 (11)	
Not involved	5 (3)	3 (2)	
Don’t know	3 (2)	2 (1)	
Relative’ satisfaction about his/her involvement	0.899
Yes, satisfied	167 (86)	154 (86)	
No, wished to be more involved	18 (9)	13 (7)	
No, wished to be less involved	1 (1)	1 (1)	
Don’t know	8 (4)	6 (3)	
Relative was told his/her loved one was likely to die	167 (86)	148 (82)	0.992
Relative was satisfied with how it was told	133 (68)	127 (71)	0.523

The number of missings varied between n=4–10 in the pre-period and n=7–16 in the post-PCP period.

* Multiple answers possible: 16 relatives in the pre-PCP group and 9 relatives in the post-PCP group gave two answers; in the post-PCP group one patient was told about the incurable disease by a nurse.

† Multiple answers possible; patients had discussed preferences for medical care with somebody else in 13 times in the pre-PCP period and 11 times in the post-PCP period.

EOLend-of-lifePCPpalliative care pathway

### Place of death and bereavement support for relatives

Two-thirds of the patients had died at their preferred place of death (66% and 58%, respectively) and the majority of relatives felt the place of death had been the right place (88% and 85%, respectively (table 3)). Around a quarter of the relatives in both groups had spoken with a hospital healthcare professional after the death of their loved ones; 18% of the relatives in the pre-PCP period and 21% of the relatives in the post-PCP period would have appreciated a conversation with a healthcare professional in the hospital after the death of their loved one. One-third of the relatives in both groups had great difficulty to cope with sorrow and to focus on other activities. The majority received (amply) sufficient help from family and friends (93% and 91%, respectively). A small percentage (10% and 8%, respectively) of the relatives had needed help or support from health and/or social services after the death of the patient (table 3). Differences between the pre- and post-PCP period were not significant and similar results were found in the per-protocol analyses.

**Table 3:** Place of death and bereavement support for relatives

	Pre-PCP (n=195)	Post-PCP (n=180)	P value
	n (%)	n (%)	
Patient died at his/her preferred place of death	129 (66)	104 (58)	0.571
On balance, relatives felt their loved one died in the right place	0.994
Yes	172 (88)	153 (85)	
No	13 (7)	12 (7)	
Not sure	7 (4)	6 (3)	
Relative had an aftercare discussion with a hospital healthcare professional regarding the death of their loved one	46 (24)	48 (27)	0.377
Relative felt this discussion was helpful	0.731
Yes	36 (78)	34 (71)	
No	4 (9)	4 (8)	
Don’t know	6 (13)	9 (19)	
Relative did not have, but would have appreciated an aftercare discussion	0.539
Yes	26 (18)	25 (21)	
No	66 (45)	58 (48)	
Don’t know	53 (36)	36 (30)	
How much effort does it take for the relative to detach from thoughts of, or grief over their loved one and focus on other possible new obligations, activities or contacts	0.028
Much effort	75 (38)	58 (32)	
Some effort	97 (50)	75 (42)	
No effort	18 (9)	37 (21)	
Relative had received support from family and friends to cope with the grief and loss of his/her loved one	0.537
Amply sufficient	119 (61)	98 (54)	
Sufficient	63 (32)	67 (37)	
Insufficient	9 (5)	4 (2)	
Relative had needed support from the health and/or social services since their loved one’s death	20 (10)	15 (8)	0.552

The number of missings varied between n=0–7 in the pre-PCP period and n=1–11 in the post-PCP period.

PCPpalliative care pathway

### Satisfaction with hospital care

The median score for general satisfaction with hospital care was 75 in both the pre-PCP and post-PCP period. Satisfaction scores were lowest for doctors’ availability, waiting time in general, hospital access and exchange of information (median scores on all four items 50 and 50, respectively). Satisfaction scores with the exchange of information were lower in the post-PCP period (p=0.042). Satisfaction scores were highest for nurses’ technical skills (75 and 71, respectively), nurses’ interpersonal skills (75 and 67, respectively) and for general satisfaction (75 and 75, respectively) (table 4). Similar results were found in the per-protocol analyses.

**Table 4:** Satisfaction with hospital care according to EORTC-IN-PATSAT32

	Scale name	Completed questions Pre-PCP n	Pre-PCP	Completed questions Post-PCP n	Post-PCP	P value
		Median (IQR)			Median (IQR)	
Doctors	Technical skills	193	67 (50–83)	169	67 (50–83)	0.388
	Interpersonal skills	192	67 (42–83)	169	67 (42–92)	0.606
	Information provision	191	67 (50–75)	168	58 (50–83)	0.642
	Availability	168	50 (38–75)	148	50 (38–75)	0.549
Nurses	Technical skills	179	75 (50–92)	148	71 (50–85)	0.567
	Interpersonal skills	182	75 (50–92)	150	67 (50–92)	0.238
	Information provision	174	58 (50–75)	149	58 (50–75)	0.398
	Availability	179	63 (50–75)	148	50 (38–75)	0.077
Other areas	Other personal interpersonal skills and information provision	182	58 (50–75)	157	58 (50–75	0.876
	Waiting time	179	50 (38–75)	161	50 (50–75)	0.451
	Hospital access	186	50 (38–75)	163	50 (38–75)	0.169
	Exchange information	174	50 (50–75)	152	50 (25–75)	0.042
	Comfort/cleanness	185	50 (50–75)	162	75 (50–75)	0.637
	General satisfaction	180	75 (50–100)	159	75 (50–75)	0.326

EORTCEuropean Organisation for Research and Treatment of Cancer

## Discussion

This study evaluated the effect of implementing a PCP on bereaved relatives’ experiences of communication and their satisfaction with EOL care. We found that bereaved relatives reported quite positively about communication and satisfaction with care even before implementation, and that their experience did not further improve after implementation of the PCP.

In the pre-PCP period, 92% of all patients had been told they had an incurable illness and 82% had discussed their preferences for EOL treatment. In our study, communication practices in the pre-PCP period were comparable to practices after interventions to improve EOL or ACP conversations in several randomised controlled trials.^24^,^26^ Timely discussing patients’ prognosis and EOL issues, preferably in the presence of a relative, is considered an important element of ACP and high-quality palliative care.^1 2^ In our study, EOL discussions mostly took place in the presence of a relative (about 85% in both groups) and for a third of the patients this occurred at least 1 year before death. Most relatives were pleased with their involvement in decision-making about their loved one’s care in both periods. In other studies, diagnosis and EOL issues are less often explicitly addressed, and also less frequently discussed in the presence of a relative.^27 28^ In a survey in seven countries of physicians’ intentions regarding discussing prognosis with terminally ill patients with cancer and their relatives, training in palliative care and a younger age of the physician were found to be associated with an active intention to discuss prognosis.^27^ The relatively high involvement of relatives in SDM in our study might be associated with a relatively young age of physicians at the participating departments, as well ass a pre-existing policy to promote palliative care.

To support bereaved relatives in coping with grief after the death of a loved one, an aftercare discussion with the involved healthcare professional can be helpful.^29^ In addition to closure, such discussions can also identify relatives with long-term grief disorders who should be referred to formal grief support services.^29 30^ In our study, aftercare discussions in the hospital occurred in approximately 25% of all cases in both groups; the majority of bereaved relatives were helped by these discussions. About 20% in both groups would have appreciated an aftercare discussion. A recent study of grief care focussing on support after a death in the hospital found that this care was provided ad hoc, based on the good will of individual staff members.^30^ This service should be offered routinely to optimise the quality and consistency of bereavement care.

We found that bereaved relatives were reasonably satisfied with the care as received in the pre-PCP and the post-PCP period, with a median score of 75 (range: 0–100). Bereaved relatives’ satisfaction was lowest for doctors’ availability, waiting time in general, hospital access and exchange of information. Comparison with other studies is difficult since the IN-PATSAT32 Questionnaire is generally used to assess satisfaction of care of hospitalised patients.^14 16 31^ However, since information exchange between different care settings is important for the continuity of care, especially at EOL, improvement trajectories seem to be required.^32^

### Strengths and limitations

This study has several strengths: it is the first prospective preintervention and postintervention study in the daily practice on an inpatient and outpatient clinic for oncology patients where a standardised digital PCP was implemented, supporting healthcare professionals not specialised in palliative care in providing structured palliative care and the initiation of ACP conversations. We have measured the communication process and satisfaction with care at EOL from a bereaved relatives perspective with two validated questionnaires (VOICES and IN-PATSAT32).^14 19^ Yet, the IN-PATSAT32 was validated to measure inpatients’ satisfaction with care, whereas we used it to measure bereaved relatives’ perspectives even though it has not been validated for this purpose. In future research, validation of the IN-PATSAT 32 questionnaire to assess relatives’ perspectives of care could be considered.

A limitation of the study concerns the background of the included patients and their relatives. Mainly relatives with a Catholic/Protestant religious background responded, even though we invited all bereaved relatives of patients fulfilling the inclusion criteria. We expected more diversity in their backgrounds since our hospital is situated in an urban area with a diverse population. What is perceived as high-quality palliative care may vary based on people’s religious backgrounds and cultural values.^33^ For future research more diversity is needed with participants with diverse religious and ethnic backgrounds whose primary language may not be the dominant language in the country where the study is performed.^34^

## Conclusion

Implementation of the PCP in a large teaching hospital did not improve communication, including SDM and ACP conversations, or satisfaction with care at the EOL as experienced by bereaved relatives. However, communication and quality of care were experienced as rather good before the implementation of the PCP. This suggests a pre-existing awareness of the importance of high-quality palliative care, probably created by a previously deployed policy in the hospital to promote palliative care. Exchange of information between different health care professionals remains an area of attention, as bereaved relatives were least satisfied with this aspect of the care provided.

## supplementary material

10.1136/spcare-2023-004495 online supplemental file 1

10.1136/spcare-2023-004495 online supplemental file 2

## Data Availability

The data of this study are kept by AP and are available upon reasonable request.
