# Supplementary material for: Quality of cancer treatment care before and after a palliative care pathway: bereaved relatives’ perspectives
Source: BMJ Support Palliat Care. 2023 Nov 16;14(e3):e004495. doi: 10.1136/spcare-2023-004495 (PMC11672035; doi:10.1136/spcare-2023-004495)
Supplement: online supplemental file 1 [file spcare-14-e3-s001.pdf]

Supplementary file 1: Description of the digital PCP

**Palliative Care Pathway**	Yes	No				
Button web link Pallialine.nl						
**General**						
Start date PCP	Open text box					
Name physician who started the PCP	Open text box					
Diagnosis	ICD10 list					
Explanation diagnosis which led to starting the PCP	Open text box					
Prognosis	Progressive (> months)		Progressive deterioration (weeks)		Dying phase (days)	
Criteria for entering palliative phase	Progressive disease	Deteriorating patient's condition	Severe complication of a medical treatment	No more anticancer treatment options available	Patients' wish to stop all medical treatments.	
WHO score	0: Able to carry out all normal activity without restriction	1: Restricted in strenuous activity but ambulatory and able to carry out light work	2: Ambulatory and capable of all self-care but unable to carry out any work activities; up and about more than 50% of waking hours	3: Symptomatic and in a chair or in bed for greater than 50% of the day but not bedridden	4: Completely disabled; cannot carry out any self-care; totally confined to bed or chair.	
Button ESAS in electronic health record[a]						
Button to add the patient to the multidisciplinary PCP meeting list						
Button to send notification to spiritual care						
**End-of-Life discussion**						
Is patient competent?	Yes	No				
Present at discussion	Relative(s)	Nurse(s)	Open text box			
Role of attending relatives	Partner	Child(ren)	Parent(s)	Brother(s) / sister(s)	Friend(s) / neighbour(s)	Open text box
Name attending relatives	Open text box					
Button web link RDMA brochure end-of-life discussion[b]						
Button to the Hospital documentation system: manual about bad-news conversation						
Description of the conversation about disease trajectory	Open text box					
Description of patient's concerns	Open text box					

**Treatment restrictions**	Any treatment restrictions are shown here. Double-click the most recent one to edit. Click the plus sign to add a new one					
Treatment restriction/will statement:	Open text box to document the discussion with patient and relatives					
Description of patient values and preferences for the time ahead	Open text box					
Description of relatives values and preferences for the time ahead	Open text box					
Preference of patient and relatives regarding out-of-hospital stay	Home	Nursing home	Palliative care unit or hospice			
**Actions following start of PCP**						
Adjusting medication	Yes	No	After selecting 'yes', the medication overview appears in which medication can be adjusted			
Adjusting outpatient visits to the hospital and/or appointments for diagnostics or medical interventions	Yes	No	Only what needs to be adjusted can be indicated here, which will then have to be adjusted by outpatient clinic employees			
Informing other care givers who are involved in the care of the patient?	Yes	No	If yes, note here which doctor is informed and in which manner			
Contact with the general practitioner?	Yes	Still to be scheduled	Not being able to get in touch		No need	
Button consultation Palliative Care Team						
Follow-up discussion following this end-of-life discussion	Tick box when follow-up discussion is needed. Then schedule a follow-up discussion					
Information leaflet about palliative care handed out	Tick box when handed out					
Button consultation paramedical professionals and aftercare office[c]	List of categories, is displayed only for admitted patients.					
[a] ESAS: Edmonton Symptom Assessment System<br>[b] RDMA: Royal Dutch Medical Association<br>[c] The aftercare office arranges all the care patients need after hospitalisation.						
