# Supplementary material for: Quality of cancer treatment care before and after a palliative care pathway: bereaved relatives’ perspectives
Source: BMJ Support Palliat Care. 2023 Nov 16;14(e3):e004495. doi: 10.1136/spcare-2023-004495 (PMC11672035; doi:10.1136/spcare-2023-004495)
Supplement: online supplemental file 2 [file spcare-14-e3-s002.pdf]

Supplementary file 2: Questionnaire Quality of Care - PCP

	General	Ref.nr. ☐☐☐
1	**Date of birth of your loved one?**<br>☐☐ day ☐☐ month ☐☐☐☐ year	
2	**When did your loved one die?**<br>☐☐ day ☐☐ month ☐☐☐☐ year	
3	**What was his/her nationality?**<br>☐ Dutch<br>☐ other, namely ________	
4	**What was his/ her marital status?**<br>☐ maried or living together<br>☐ widow<br>☐ divorced<br>☐ single<br>☐ other, namely ________	
5	**Did your loved one had children?**<br>☐ yes<br>☐ no	
6	**What age is this child or these children?**<br>*(you can tick multiple boxes)*<br>☐ 0-12 year number of children in this age: ☐<br>☐ 13-18 year number of children in this age ☐<br>☐ 19 years and older number of children in this age: ☐	
7	**How was your loved one's living situation?**<br>☐ single-housing<br>☐ with partner<br>☐ with partner and child / children<br>☐ without partner, with child / children<br>☐ with parent(s)<br>☐ other	
8	**What was his/her highest completed level of education?**<br>☐ primary education<br>☐ lower secondary education<br>☐ upper secondary education<br>☐ post-secondary non-tertiary education<br>☐ short-cycle tertiary education<br>☐ bachelor or equivalent<br>☐ other	

9	**Was your loved one religious or did he/she have a certain philosophy/religion of life?**<br>☐ yes<br>☐ no<br>☐ don't know
10	**Which philosophy/religion?**<br>☐ Catholic<br>☐ Protestant<br>☐ Islamic<br>☐ Buddhist<br>☐ Hindu<br>☐ don't know<br>☐ other ☐
11	**What kind of relation did you have with your loved one?**<br>☐ partner<br>☐ mother<br>☐ father<br>☐ child<br>☐ other ☐
12	**What is your age?**<br>☐☐ year
13	**Are you a woman or a man?**<br>☐ man<br>☐ woman
14	**How is your health in general?**<br>☐ very good<br>☐ good<br>☐ average<br>☐ good days / bad days<br>☐ bad

	**The following questions are concerned with information that the health professionals may have given you and………….. about his/ her illness, and about any anxiety or depression he/she may have had**
15	**During his/her last three months, did…………………… suffer from anxiety and/or nerves or get depressed? Please tick one box**<br><br>☐ yes, most of the time<br>☐ yes, often

	☐ yes, sometimes<br>☐ rarely<br>☐ never<br><br>**b) If Yes: Did he/she get any help to cope with these problems?**<br><br>☐ yes ☐ no ☐ help was not necessary<br><br>**c) If yes, did they help relieve the anxiety, nerves or depression?**<br><br>☐ yes, most of the time<br>☐ yes, often<br>☐ yes, sometimes<br>☐ rarely<br>☐ never<br>**Please comment if you would like to:** ☐
**16**	**a) During ……………'s illness or incapacity were you able to find out all you wanted to know about his or her illness and how it was likely to affect him / her?**<br>☐ yes ☐ no<br><br>**b) Was there anything else you would like to have been explained to you in more detail?**<br>☐ yes ☐ no<br><br>**If yes, please say which things you would liked to have known more about:**<br>☐
**17**	**How involved were you with the decisions made about……. 's care?**<br>☐ very involved<br>☐ fairly involved<br>☐ not involved<br>☐ don't know
**18**	**Were you satisfied with your involvement?**<br>☐ no, I wished to be more involved<br>☐ no, I wished to be less involved<br>☐ yes<br>☐ don't know
**19**	**Were you told he/she was likely to die?**<br>☐ yes ☐ no<br><br>**If yes, were you satisfied with the way you were told?**<br>☐ yes ☐ no

	**Please comment if you would like to:**<br>[ ]<br><br>**If no, would you have liked to have been told?**<br>☐ yes ☐ no<br><br>**Please comment if you would like to:**<br>[ ]
	**The following questions are about the circumstances surrounding ……..'s death, and your feelings about the way in which the health and social services treated you and……………. at that time.**
**20**	**Where did….. die?**<br>☐ his/her own home<br>☐ your homes<br>☐ hospital<br>☐ hospice<br>☐ old people's home / nursing home<br>☐ on the way to hospital<br>☐ on the way to hospice<br>☐ other (please explain) [ ]
**21**	**a) Did……. ever say that there was a place where he/she would like to die?**<br>☐ yes<br>☐ no<br>☐ don't know<br><br>**b) If yes, where was the place?**<br>☐ at home<br>☐ In an old people's home / nursing home<br>☐ In a hospice<br>☐ In a hospital<br>☐ other (please explain) [ ]

22	**Did he/she died in the place he/she wanted to die in?**<br>☐ yes<br>☐ no<br>☐ don't know<br><br>**If no, what was the reason for that? Please tick one box only**<br>☐ he/she changed his mind<br>☐ there was insufficient help for him/her to stay at home<br>☐ the family/carers were too tired to continue looking after him/her at home<br>☐ the staff at the old peoples/nursing home were no longer able to look after him/her<br>☐ he/she could no longer afford to stay there<br>☐ he / she deteriorated and dies so quickly that there wasn't time for him/her to be moved<br>☐ there were no beds available<br>☐ other (please explain)
23	**On balance. Do you feel that where he dies was the right place for him or not?**<br>☐ Yes, it was the right place<br>☐ No, it wasn't the right place<br>☐ Not sure<br><br>**If no, was it because**<br>☐ It wasn't where he/ she wanted to die<br>☐ The care he / she received there was poor<br>☐ It was too far away from family and friends<br>☐ Other (please explain)
24	**Is there any other help or support you would have liked from the health and/or social services since his/her death?**<br>☐ yes ☐ no<br><br>**If yes, please comment on what you feel would have helped**

	**Shared decision-making End-of-life**
25	**Was your loved one told he/she had an incurable illness?**<br>☐ Yes, by a medical specialist<br>☐ Yes by a ward physician<br>☐ Yes, by a family doctor or physician in a nursing home<br>☐ Yes, all above mentioned<br>☐ no
26	**Were you present during this message/discussion?**<br>☐ yes<br>☐ no<br>☐ other

27	**How long before death of your loved one's death was told about this incurable illness?**<br>☐ More than 12 months before death<br>☐ 3-12 months before death<br>☐ 1 week – 3 months before death<br>☐ Less than a week before death
28	**When this message was told to your loved one and/or you, were you given the opportunity to talk about it?**<br>☐ yes<br>☐ no<br>☐ don't know<br><br>**Please comment if you would like to:** ☐
29	**Before his/her death, did your loved one discuss with someone his/her preferences for end-of-life medical treatment?** *(You can tick multiple boxes)*<br>☐ yes, with:<br>☐ partner<br>☐ children<br>☐ other family members<br>☐ friends<br>☐ family doctor<br>☐ medical specialist<br>☐ physician in a nursing home<br>☐ nurse<br>☐ somebody else ☐<br>☐ no
30	**If yes, do you know which preferences were discussed?**<br>☐ yes<br>☐ no<br>**If yes, such as:** ☐
31	**Did they fulfill these preferences?**<br>☐ yes<br>☐ no<br>**If no, please explain:** ☐
32	**Do you think if your loved one had needed more discussions regarding his or her preferences for end-of-life medical treatment?**<br>☐ yes<br>☐ no<br>☐ don't know
33	**If yes, do you know about which preferences your loved one wanted to discuss?**<br>☐ yes<br>☐ no ☐

	**If yes, such as:**
**34**	**Do you think your loved one was sufficiently involved in his or her medical treatment?**<br>☐ yes<br>☐ sometimes<br>☐ no<br>☐ don't know<br>**Please explain**
**35**	**Was your loved one able to handle and complete issues with related to his or her imminent death?**<br>*It includes other issues than medical discussions, e.g. doing things from their bucket list, recovering lost contacts, arranging funeral and/or legacy, saying goodbye, etc.*<br>☐ yes<br>☐ no<br>**Please explain**

Satisfaction with care in the Maasstad Hospital (out- and / or inpatient)

	How would you rate doctors, in terms of:	Poor	Fair	Good	Very good	Excellent
**36**	Their knowledge and experience of your illness?	1	2	3	4	5
**37**	The treatment and medical follow-up they provided?	1	2	3	4	5
**38**	The attention they paid to your physical problems?	1	2	3	4	5
**39**	Their willingness to listen to all of your concerns?	1	2	3	4	5
**40**	The interest they showed in you personally?	1	2	3	4	5
**41**	The comfort and support they gave you?	1	2	3	4	5
**42**	The information they gave you about your illness?	1	2	3	4	5
**43**	The information they gave you about your medical tests?	1	2	3	4	5
**44**	The information they gave you about your treatment?	1	2	3	4	5
**45**	The frequency of their visits/consultations?	1	2	3	4	5
**46**	The time they devoted to you during visits/consultations?	1	2	3	4	5

How would you rate nursus (if applicable), in terms of:	Poor	Fair	Good	Very good	Excellent	N.a.

**47**	The way they carried out your physical examination (took your temperature, felt your pulse,...)?	1	2	3	4	5	N.a
**48**	The way they handled your care (gave your medicines, performed intravenous injections,...)?	1	2	3	4	5	N.a
**49**	The attention they paid to your physical comfort?	1	2	3	4	5	N.a
**50**	The interest they showed in you personally?	1	2	3	4	5	N.a
**51**	The comfort and support they gave you?	1	2	3	4	5	N.a
**52**	Their human qualities (politeness, respect, sensitivity, kindness, patience,...)?	1	2	3	4	5	N.a
**53**	The information they gave you about your medical tests	1	2	3	4	5	N.a
**54**	The information they gave you about your care?	1	2	3	4	5	N.a
**55**	The information they gave you about your treatment?	1	2	3	4	5	N.a
**56**	Their promptness in answering your buzzer calls?	1	2	3	4	5	N.a
**57**	The time they devoted to you?	1	2	3	4	5	N.a
**How would you rate services and care organisation of the hospital in general?**		**Poor**	**Fair**	**Good**	**Very good**	**Excellent**	**N.a.**
**58**	The exchange of information between caregivers?	1	2	3	4	5	N.a
**59**	The kindness and helpfulness of the technical, reception, laboratory personnel,...?	1	2	3	4	5	N.a
**60**	The information provided on your admission to the hospital?	1	2	3	4	5	N.a
**61**	The information provided on your discharge from the hospital?	1	2	3	4	5	N.a
**62**	The waiting time for obtaining results of medical tests?	1	2	3	4	5	N.a
**63**	The speed of implementing medical tests and/or treatments?	1	2	3	4	5	N.a
**64**	The ease of access (parking, means of transport,...)?	1	2	3	4	5	N.a
**65**	The ease of finding ones way to the different departments?	1	2	3	4	5	N.a.
**66**	The environment of the building (cleanness, spaciousness, calmness,...)?	1	2	3	4	5	N.a.
**67**	Overall, how would you rate the care received during your hospital stay?	1	2	3	4	5	N.a.

	Post-bereavement needs
68	**After the death of your loved one, have you spoken with a hospital healthcare professional regarding his/her illness or death? (***You can tick multiple boxes)*<br>☐ yes, directly after his / her death<br>☐ yes, after a few weeks<br>☐ no
69	**If yes, have these discussion(s) helped you?**<br>☐ yes<br>☐ no<br>☐ don't know<br>**Please explain**
70	**If no, would you have appreciated talking to someone?**<br>☐ yes<br>☐ no<br>☐ don't know
71	**How much effort did it take to detach yourself from thoughts of, or grief over your loved ones and focus on other possible new obligations, activities or contacts?**<br>☐ great difficulty<br>☐ some difficulty<br>☐ no difficulty
72	**Have you received sufficient support from family and friends to cope with the grief and loss of your loved one?**<br>☐ yes, amply sufficient<br>☐ yes, sufficient<br>☐ no, insufficient
73	***If you might have a final remark concerning coping with the loss of your loved one, please explain it below:***
